# Supplementary material for: Differences and Similarities between Front-of-Pack Nutrition Labels in Europe: A Comparison of Functional and Visual Aspects
Source: Nutrients. 2019 Mar 14;11(3):626. doi: 10.3390/nu11030626 (PMC6471039; doi:10.3390/nu11030626)
Supplement: Supplementary file 1 [file nutrients-11-00626-s001.pdf]

Supplementary materials:

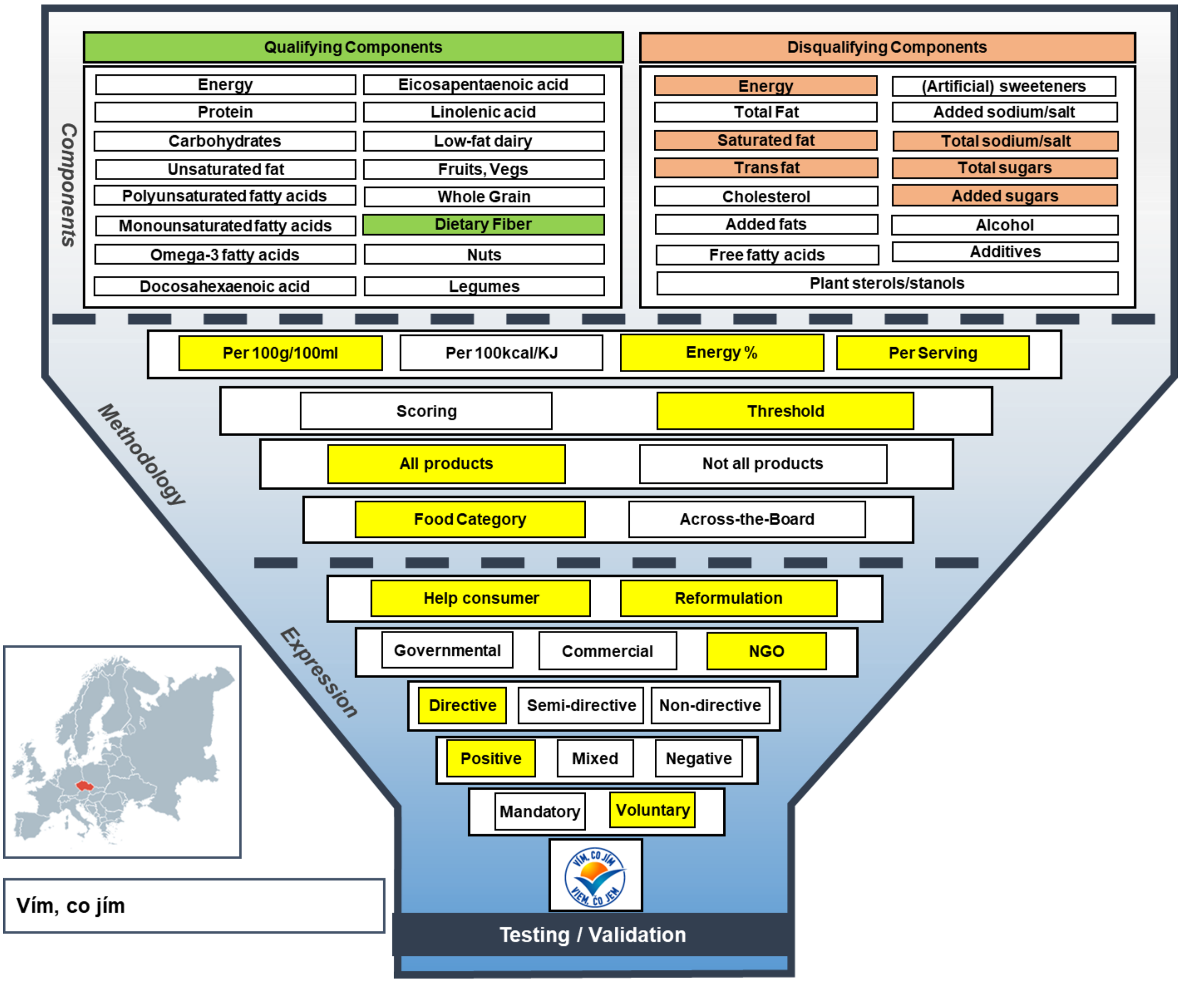

Figure S1. Characteristics of Czech Choices label 'Vim, co Jim' shown in the Funnel Model.

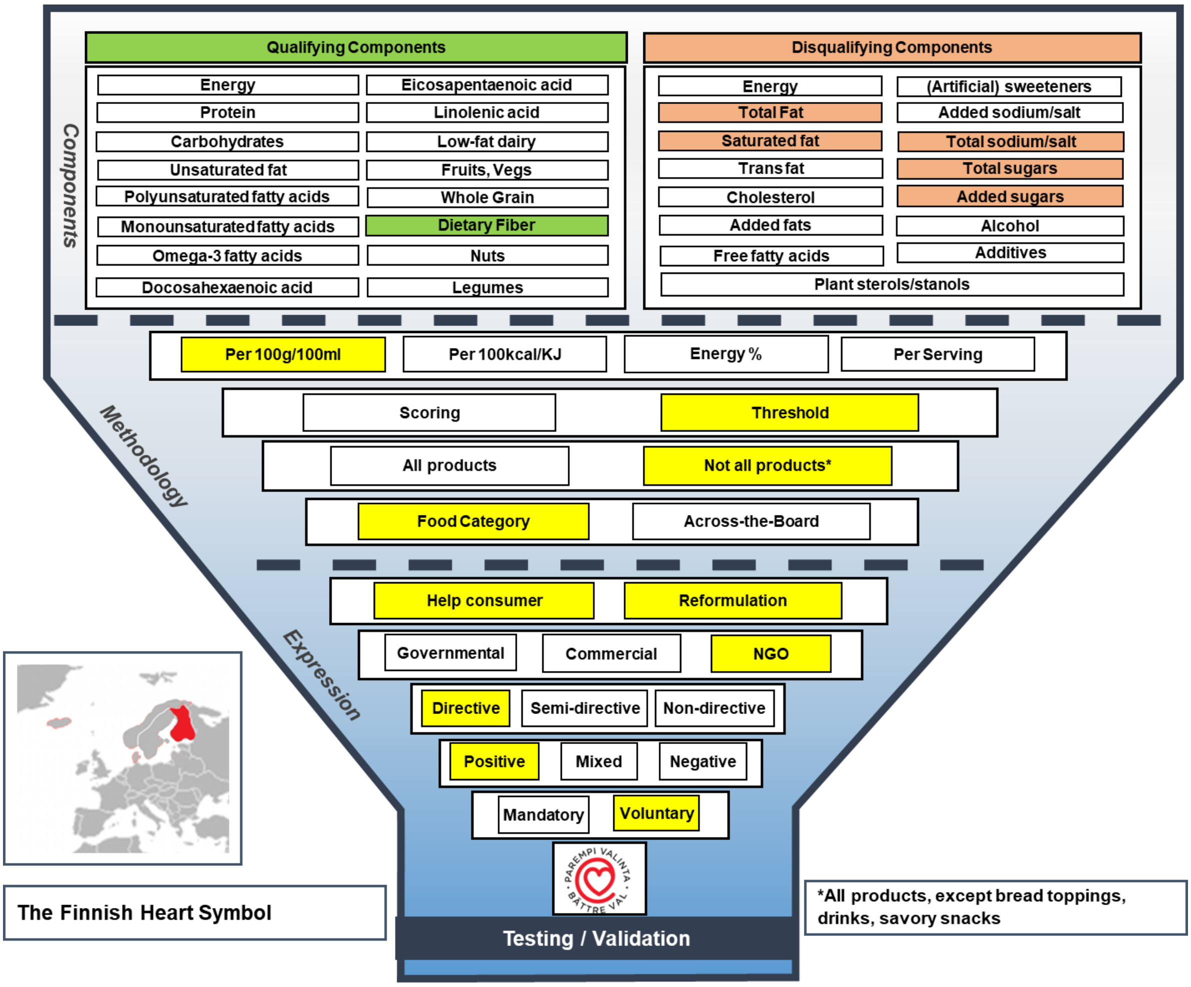

Figure S2. Characteristics of the Finnish Heart label shown in the Funnel Model.

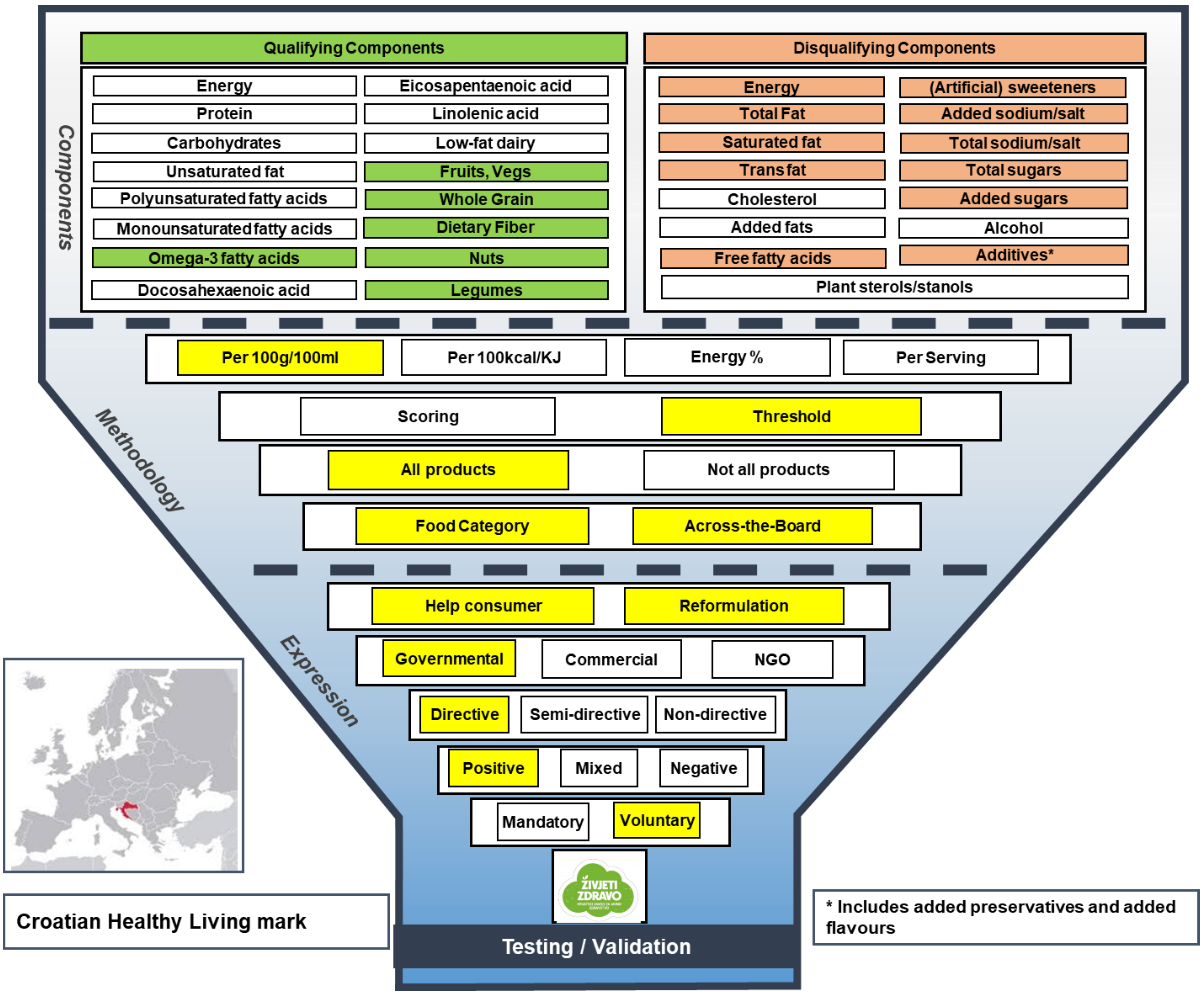

Figure S3. Characteristics of the Croatian Healthy Living (HL) label shown in the Funnel Model.

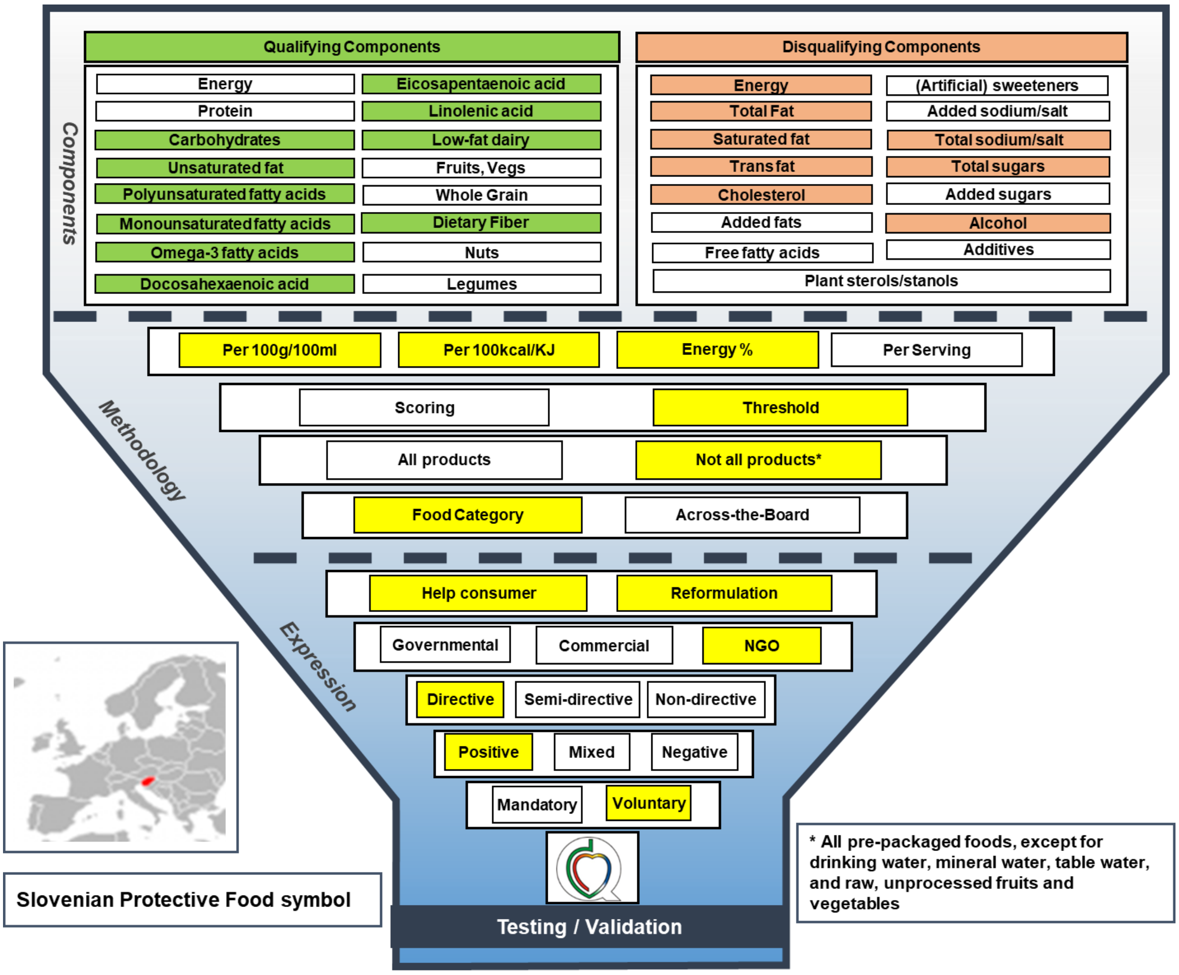

Figure S4. Characteristics of the Slovenian Protective Food (PF) label shown in the Funnel Model.

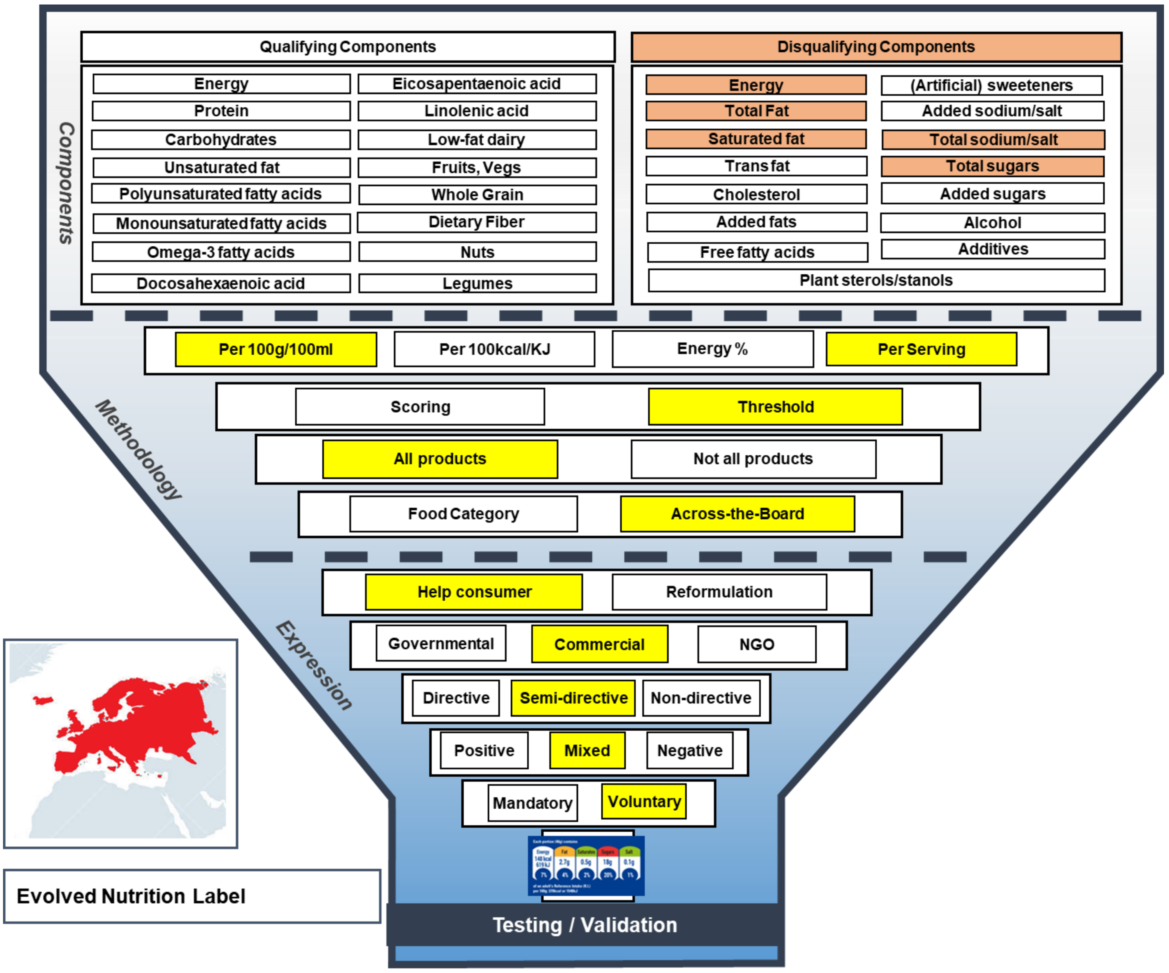

Figure S5. Characteristics of the Evolved Nutrition Label (ENL) shown in the Funnel Model.

Table S1. Qualifying and disqualifying components used in positive front-of-pack (FOP) labelling systems in Europe.

	Choices	Vim, co jim	Keyhole	Finnish Heart	Croatian Healthy Living	Slovenian Protective Food
Qualifying components						
Energy	-	-	x	-	-	-
Carbohydrates	-	-	-	-	-	x
Unsaturated fat	-	-	-	-	-	x
Monounsaturated fatty acids (MUFA)	-	-	-	-	-	x
Polyunsaturated fatty acids (PUFA)	-	-	-	-	-	x
Omega-3 fatty acids (W-3)	-	-	-	-	x	x
Eicosapentaenoic acid (EPA)	-	-	-	-	-	x

Docosahexaenoic acid (DHA)	-	-	-	-	-	x
Linolenic acids	-	-	-	-	-	x
Low-fat dairy	-	-	-	-	-	x
Fruits & vegetables	-	-	x	-	x	-
Whole grain	-	-	x	-	x	-
Dietary fibre	x	x	x	x	x	x
Nuts	-	-	-	-	x	-
Legumes	-	-	x	-	x	-
Disqualifying components						
Energy	x	x	x	-	x	x
Total fat	-	-	x	x	x	x
Saturated fat (SFA)	x	x	x	x	x	x
Trans fat (TFA)	x	x	x	-	x	x
Cholesterol	-	-	-	-	-	x
Plant sterols/ stanols	-	-	x	-	-	-
Added fats	-	-	x	-	-	-
(Artificial) sweeteners	-	-	x	-	x	-
Total salt/ sodium	x	x	x	x	x	x
Added salt/ sodium	-	-	-	-	x	-
Total sugar	x	x	x	x	x	x
Added sugar	x	x	x	x	x	-
Additives	-	-	-	-	x	-
Alcohol	-	-	-	-	-	x
Free fatty acids	-	-	-	-	x	-

Table S2. Qualifying and disqualifying components used in mixed and negative front-of-pack (FOP) labelling systems in Europe.

	Multiple Traffic Light	Nutriscore	Israeli warning
Qualifying components			
Protein	-	x	-
Fruits & vegetables	-	x	-
Dietary fibre	-	x	-
Nuts	-	x	-
Legumes	-	x	-
Disqualifying components			
Energy	x	x	-
Total fat	x	-	-
Saturated fat (SFA)	x	x	x
Total sodium/ salt	x	x	x
Total sugars	x	x	x
